# Efficacy of ankle Kinesio taping on balance and functional performance in obese female collegiate students: a prospective randomized placebo-controlled trial

**DOI:** 10.3389/fpubh.2025.1631757

**Published:** 2025-08-11

**Authors:** Nesma M. Allam, Hadaya Mosaad Eladl, Dalia Mahmoud Abdelmonem Elsherbini, Moaz Abulfaraj, Moneer E. Almadani, Ateya Megahed Ibrahim, Mohamed El-Sherbiny, Mohamed A. Eladl, Nermine Nosseir, Reda Jamjoom

**Affiliations:** ^1^Department of Physical Therapy and Health Rehabilitation, College of Applied Medical Sciences, Jouf University, Sakaka, Saudi Arabia; ^2^Department of Clinical Laboratory Sciences, College of Applied Medical Sciences, Jouf University, Sakaka, Saudi Arabia; ^3^Department of Surgery, Faculty of Medicine, King Abdulaziz University, Jeddah, Saudi Arabia; ^4^Department of Clinical Medicine, College of Medicine, AlMaarefa University, Riyadh, Saudi Arabia; ^5^College of Nursing, Prince Sattam bin Abdulaziz University, Al-Kharj, Saudi Arabia; ^6^Department of Family and Community Health Nursing, Faculty of Nursing, Port Said University, Port Said, Egypt; ^7^Department of Basic Medical Sciences, College of Medicine, AlMaarefa University, Diriyah, Saudi Arabia; ^8^Department of Basic Medical Sciences, College of Medicine, University of Sharjah, Sharjah, United Arab Emirates; ^9^Department of Biomedical Sciences, College of Medicine, Gulf Medical University, Ajman, United Arab Emirates

**Keywords:** Kinesio taping, balance, postural stability, functional performance, obese females

## Abstract

**Objective:**

This study aimed to evaluate the efficacy of ankle Kinesio taping (KT) on static and dynamic balance and functional performance in obese female collegiate students.

**Design:**

This study was a prospective, randomized controlled trial.

**Setting:**

The research was conducted in outpatient physical therapy settings.

**Participants:**

A total of 40 female collegiate students, aged 18–25 years, were randomly assigned to two groups of equal size.

**Intervention:**

Group A (KT group) received KT with 75–100% tension, and group B (Placebo KT group) received placebo KT with 0% tension. KT was applied to the dominant leg in both groups for 6 weeks.

**Outcome measures:**

The primary outcome was static balance, which was measured using the single-leg stance test (SLST). The secondary outcomes were dynamic balance, assessed using the Biodex Balance System (BBS), and functional performance, which was assessed using the single-leg vertical jump test (SLVJT) and single-leg hop test (SLHT). All outcomes were assessed at baseline and after 6 weeks.

**Results:**

The KT group showed a statistically significant increase in SLS, SLVJT, and SLHT (*p* < 0.001), compared to the placebo KT group. In contrast, a statistically significant decrease was observed in the OSI, MLSI, and APSI (*p* < 0.001) in the KT group compared to the control group after a period of 6 weeks.

**Conclusion:**

The application of a 6-week KT mechanical correction technique can improve static and dynamic balance and functional performance in obese female university students compared to the placebo group. Including KT in the treatment plan is recommended for obese women with balance and functional performance deficits.

**Clinical trial registration:**

ClinicalTrials.gov, identifier NCT06195748.

## Introduction

Obesity is defined as a body mass index (BMI) greater than 30 kg/m^2^ (1). It is a complex and chronic disease that affects women and older adults, regardless of their geographic region, social status, or economic status (2). Excess body mass may increase the risk of deterioration and injury to musculoskeletal structures due to repeated stress during weight-bearing activities (3). Furthermore, during the stance phase, the tibiotalar joints can withstand stress up to five times a person’s body weight, and this stress is further intensified in obese individuals (4).

Excessive weight is one of the factors that cause ankle sprains, anatomical misalignment, postural fluctuation, reduction in muscle power and reaction time, and relaxation of the ankle joint (5). Ankle joint relaxation is influenced by sex, with women experiencing a higher incidence of lateral ankle ligament injury in females compared to men. Furthermore, estrogen produced during the menstrual cycle causes excessive laxity in joints and muscles (6). This increased laxity limits their ability to control dynamic motion, which explains the higher risk of musculoskeletal injuries in women (7).

Kinesio taping (KT) is a method that involves applying a flexible, adhesive cotton tape to the skin. Kinesio tape is non-allergenic, thin, and lightweight; therefore, it can be perceived as a part of the body. It can be stretched by 140% of its original length and maintained in position for 3–5 days without causing any negative consequences (8). Kinesio taping enhances proprioceptive feedback and stimulates neurological responses and somatosensory function in superficial mechanoreceptors (9). It has been proposed that the facilitatory impact and mechanical properties of ankle braces might improve postural control, increase ankle joint performance, and reduce ankle instability (10).

Kinesio taping has been shown to enhance balance in basketball players with functional ankle instability (FAI) (10, 11), in healthy subjects (12), and in those with multiple sclerosis (13). Moreover, it may enhance functional performance in participants with and without ankle injuries (14). Limited research has evaluated the efficacy of ankle KT on static and dynamic balance and functional performance in obese female university students. The findings of this study would help clinicians in making decisions about the use of KT in obese adult women when improvement in balance and functional performance is desired.

## Materials and methods

### Study design

This prospective, randomized, double-blind, placebo-controlled study was conducted at the physical therapy laboratories of the College of Applied Medical Sciences, Jouf University, from January to September 2024. The study protocol adhered to the CONSORT standards for randomized trials of alternative treatments. All procedures were approved by the Research Ethics Committee of Qurayyat Health Affairs (IRB-No. 2023-124). This study was registered at ClinicalTrials.gov (No. NCT06195748) in accordance with the principles of the Declaration of Helsinki.

### Participants

A total of 40 female university students were recruited based on the following inclusion criteria: aged between 18 and 25 years and a BMI of 30–39.9 kg/m^2^. The exclusion criteria included any orthopedic or neurological injuries in the past 6 months, edema of the ankle joint, skin sensitivity to KT, auditory/vestibular disorders that compromised balance, visual problems, participants suffering from diabetes mellitus, open wounds, inability to follow instructions due to personal or cognitive problems, and regular physical training in the last 3 months. Participants were equally divided into two groups: Group A (KT group) received KT with 75–100% tension, and Group B (Placebo KT group) received KT with 0% tension; both KT techniques were applied to the dominant leg for 6 weeks. Before participating in the study, the authors explained the procedures, and each participant provided signed informed consent.

### Sample size

The sample size was calculated using G-Power software (version 3.1.9.2; Franz Faul, Universität Kiel, Germany) according to the single-leg test with the following parameters: α = 0.05, effect size = 0.25, and β = 0.2. The present analysis had an actual power of 80% (1-β), with each group comprising a minimum of 18 subjects. This original estimate was adjusted to 40 participants in both groups, with a 10% attrition rate (12).

### Randomization

Forty participants were randomly divided into Group A (KT group, *n* = 20) and Group B (placebo KT group, *n* = 20). The block randomization program was generated by a computer at http://www.randomization.com/. To minimize bias and group variability, participants were randomly allocated to blocks 4, 6, and 8 using a 1:1 allocation ratio. Randomization was conducted by a single author who did not participate in recruitment, data gathering, or treatment. To ensure concealed allocation, randomization codes were consecutively labeled and kept confidential in concealed opaque envelopes. After the baseline assessment, the independent investigator opened the next envelope sequentially to reveal the group assignment and directed the taping procedure accordingly.

### Blinding

This study was double-blinded. The participants were blinded to group allocation using a placebo taping method that visually resembled the Kinesio taping intervention but did not apply tension. Both the intervention and placebo tapes were applied to the same anatomical region by a certified physical therapist trained in both techniques. The outcome assessors were blinded to group assignments. All balance and functional performance assessments were conducted by a separate team that was not involved in the intervention procedures and was unaware of the participants’ group assignment. Group codes were used during data entry and analysis to maintain the blinding of the data analyst until the statistical analysis was completed.

### Procedures

A certified orthopedic physical therapist conducted a pre-participation orthopedic ankle examination during the first interview with the participants. Patients with congenital and/or neurological abnormalities that might have affected the experimental data were excluded. Ligamentous stability of the ankle was assessed using a stress test, and cutaneous sensation was also examined. The dominant leg was determined using a ball-kick test. The weight of each participant was assessed twice a week in conjunction with KT application to ensure that no weight loss occurred during the study period. Ten minutes of warm-up techniques in the form of jumping, jogging, squatting, and submaximal kicking were performed (15).

### Intervention

#### Study group

**Figure 1:**
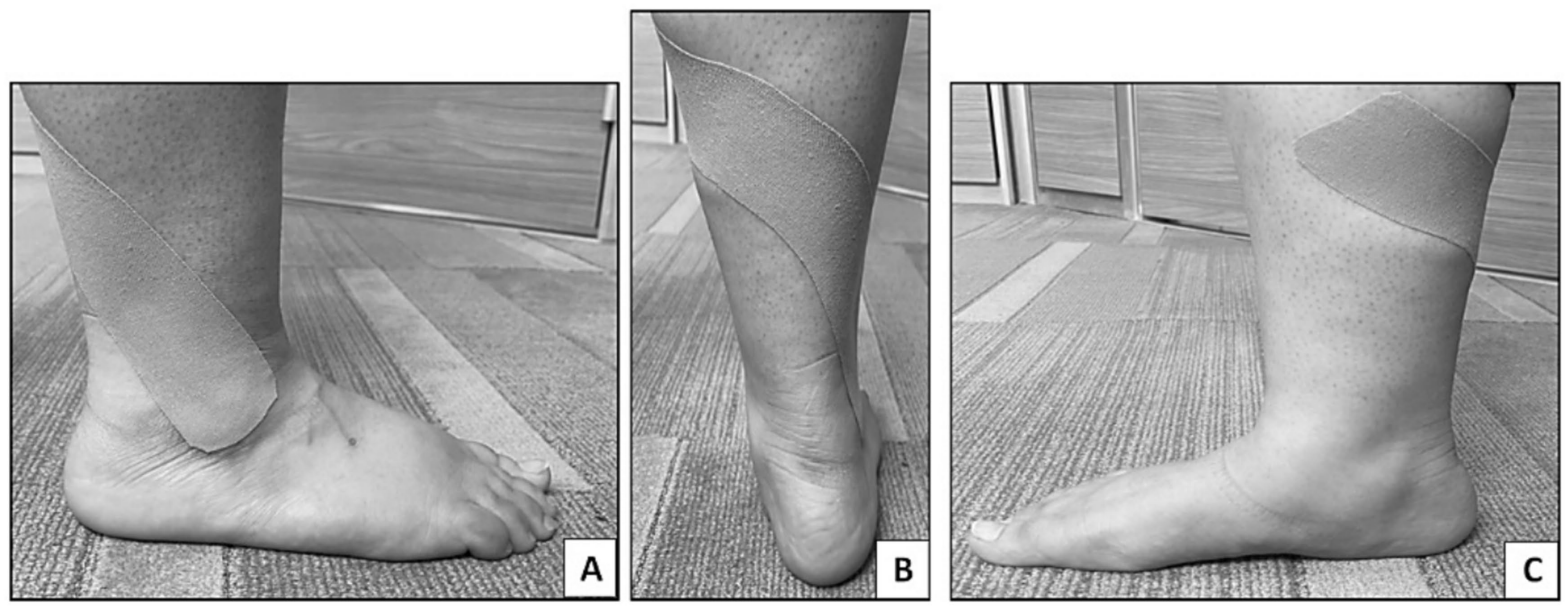
Kinesio tape application on the dominant leg to group A (KT group). **(A)** Starting application of KT at the lateral malleolus, **(B)** application 75–100% tension toward the posterior aspect of the leg, **(C)** continue the tension of KT till the medial aspect of the tibia (middle 1/3).

Following the approach suggested by Kase et al. (16), waterproof flexible adhesive tape (thickness of 0.5 mm and width of 5 cm) was applied. The posterosuperior mechanical correction glide technique was applied manually to the lateral malleolus of the dominant leg of the participants. Individuals were asked to maintain a standing position with no footwear in a neutral position on a 30 cm high tool. An I-shaped KT (20 cm in length) was applied starting at the lateral malleolus and directed toward the tibia (middle 1/3). To enhance sensation and improve motion, a tension of 75–100% is proposed, as it provides guidance to the ankle joint into a more functional alignment, and higher tension resists excessive inversion or plantarflexion, which are common injury mechanisms for the ankle (16) (Figure 1). After application, maximum tape adhesion was achieved by the therapist stroking the hand along the length of the wide tape three times (12). For consistency, the same procedure was performed by the same practitioner for all participants.

#### Control group

**Figure 2:**
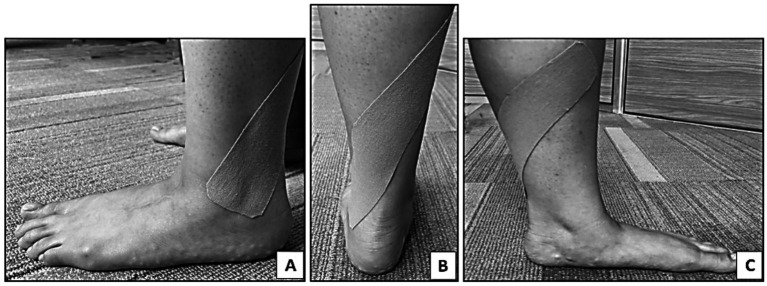
Kinesio tape application on the dominant leg to group B (Placebo KT group). **(A)** Starting application of KT at the lateral malleolus, **(B)** apply 0% tension toward the posterior aspect of leg, **(C)** continue application without tension till the medial aspect of tibia (middle 1/3).

In the placebo control group, KT was applied in the same manner as in the experimental group, but with a longer tape (28 cm) with no tape tension or fibular glide (Figure 2). Placebo taping helped distinguish between true physiological effects and psychological or perceptual changes (12).

In both groups (experimental and placebo), the tape was replaced every 4th–5th day until the end of the study (16). Nevertheless, patients were asked to record any experience of pain or discomfort during the interaction with the physiotherapist.

#### Outcome measures

The main outcome was static balance, which was evaluated using the single-leg stance test (SLST). The secondary outcome measures were dynamic balance, assessed using the Biodex Balance System (BBS), and functional performance, assessed using the single-leg vertical jump test (SLVJT) and single-leg hop test (SLHT). All tests were separated by one-minute rest periods. All participants were evaluated by a blinded examiner at baseline and after 6 weeks.

#### Static balance

Static balance was evaluated using the SLST. The participants were instructed to stand barefoot on the dominant lower limb with their eyes closed and their hands folded across their chests. The foot of the non-dominant leg was elevated until it was close to the ankle of the stance leg, but without touching it. A stopwatch was used to measure the duration for which the individual was able to stand on one limb in seconds, starting when the participant lifted the foot off the ground and terminating when the participant (1) uncrossed his arms, (2) moved the non-dominant limb close to or far from the dominant leg, or (3) lowered it to contact the ground to maintain balance. (4) After 45 s, or (5) the eyes that had been closed were opened. The average of the three test repetitions was calculated (17). The Intraclass Correlation Coefficient (ICC) for this test was 0.998 for closed eyes, indicating excellent intra-rater reliability (18).

#### Biodex Balance System

The Biodex Balance System (Biodex Medical Systems Inc., Shirley, NY, United States) was used to evaluate the postural stability. It showed an intra-rater reliability of (ICC = 0.83). Each participant was instructed to stand with their upper limbs on their sides and to maintain balance. With a 10-s break between trials, the overall stability index (OSI), mediolateral stability index (MLSI), and anteroposterior stability index (APSI) were measured (19). The first step in the test was to enter the required data and test parameters into the Biodex software. Before the main test, the participants were asked to stand barefoot on both legs, with their eyes open, on a medium level of difficulty (level 5), with a 30-s trial period and a 10-s rest period. They also had to complete one familiarization trial to learn how to keep the platform as horizontal as possible using screen visual feedback, and the platform was released after a 5-s delay from pressing the start key (20).

#### Single-leg vertical jump test

The objective of the SLVJT is to land on the floor with the dominant leg after jumping as high as possible from a single foot. The procedures for the SLVJT included the following: (1) The participants stood unsupported on one leg, close to a wall, tapped their palm on the wall at the highest possible vertical point, and then a reference mark was made on the wall by their index finger. (2) At take-off, the participants jumped as far as they could, tapped their hand on the wall at the highest vertical point, landed on the same lower limb, and marked their position on the wall. (3) Jump displacement was measured in centimeters as the difference between the standing reach height and peak jump height. The participants were instructed to use a chosen countermovement without stepping and to freely swing their arms before jumping. The average of the two best outcomes was calculated using three attempts (21). The SLVJ test has high reliability with an ICC of 0.93 (22).

#### Single leg hops test

The aim of the SLHT was to jump as far as possible on one foot and land on the same foot while maintaining balance. The SLHT procedure included the following: (1) All participants placed their big toe on a predefined mark on the floor while standing on their dominant leg. (2) The participants landed on the same extremity as far forward as possible after the hop. (3) The participants maintained their landing for at least 2 s. The assessor recorded the horizontal displacement, in centimeters, between the big toe starting point and the heel landing mark using a conventional tape measure. Before jumping, participants were instructed to use a selected countermovement without stepping and to swing their arms freely. The average of the two best performances from the three trials was recorded (23). The ICC of the SLHT was 0.93 (22).

#### Statistical analysis

GraphPad Prism version 9 was used to analyze the data, which were displayed as the mean ± SD. The difference in demographic characteristics between the KT and placebo KT groups was determined using Student’s *t*-test. A two-way repeated-measures ANOVA was used to evaluate the outcome variables between the groups. Qualitative data are presented as percentages. We used the chi-square test for comparison between variables. Partial eta square was used to evaluate the effect size between groups. Statistical significance was set at *p <* 0.05. A paired *t*-test was used to evaluate the differences within each group. After standardizing the data, all extremes were eliminated. The relationship between SLVJT, SLHT, and OSI post-intervention in both groups was assessed using a linear regression model with a generalized estimating equation (GEE) adjustment. The normality and homoscedasticity of the variance were statistically evaluated prior to using parametric assumptions. *Post-hoc* power analysis for clinical parameters was conducted using MedCalc Software Ltd. (Version 23.2.3) (24) to estimate power for comparison of means between the KT and placebo KT groups post-intervention.

## Results

**Figure 3:**
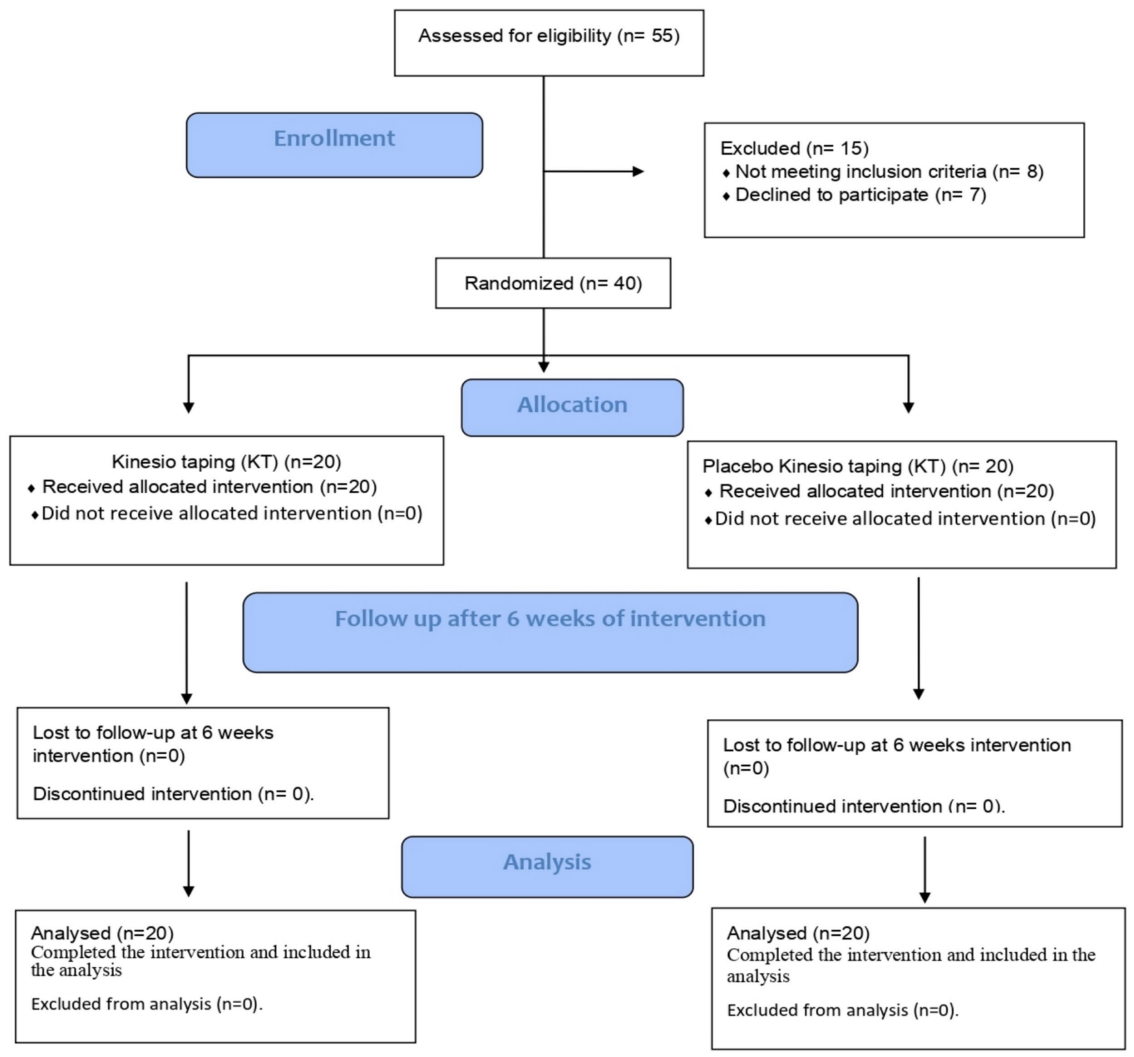
Flowchart of ankle Kinesio taping clinical study.

Fifty-five participants were enrolled in the study. Fifteen patients were excluded: eight did not meet the inclusion criteria, and seven refused to participate (Figure 3). All participants completed the intervention and were included in the statistical analyses.

### Subject characteristics

Table 1 presents the characteristics of the study and control groups of patients. The mean age, weight, height, BMI, and dominant limb did not differ significantly between the two groups (*p* > 0.05).

**Table 1:** Baseline demographic data of the subjects (*N* = 40).

Characteristics	Mean ± SD		MD		*p*	95% CI	
	KT (*N* = 20)	Placebo KT (*N* = 20)		Effect size (η^2^)		Lower	Upper
Age (years)	21.40 ± 2.03	21.23 ± 2.03	−0.18	0.002	0.99 (ns)	−1.48	1.13
Weight (Kg)	86.83 ± 10.52	87.70 ± 7.64	0.88	0.002	0.77 (ns)	−5.01	6.76
Height (cm)	160.2 ± 5.67	160.5 ± 2.82	0.30	0.001	0.83 (ns)	−2.57	3.17
BMI (kg/m^2^)	33.87 ± 3.67	34.11 ± 3.32	0.24	0.001	0.83 (ns)	−2.00	2.48
Dominant limb							
Right	17 (85%)	16 (80%)					
Left	3 (15%)	4 (20%)					
Χ^2^ (*p*-value)	0.17 (0.68)						

N, number; BMI, Body mass index, mean ± SD; *p* ≥ 0.05, statistically non-significant (ns); *Χ*^2^, Chi-square test.

### Clinical measures

**Figure 4:**
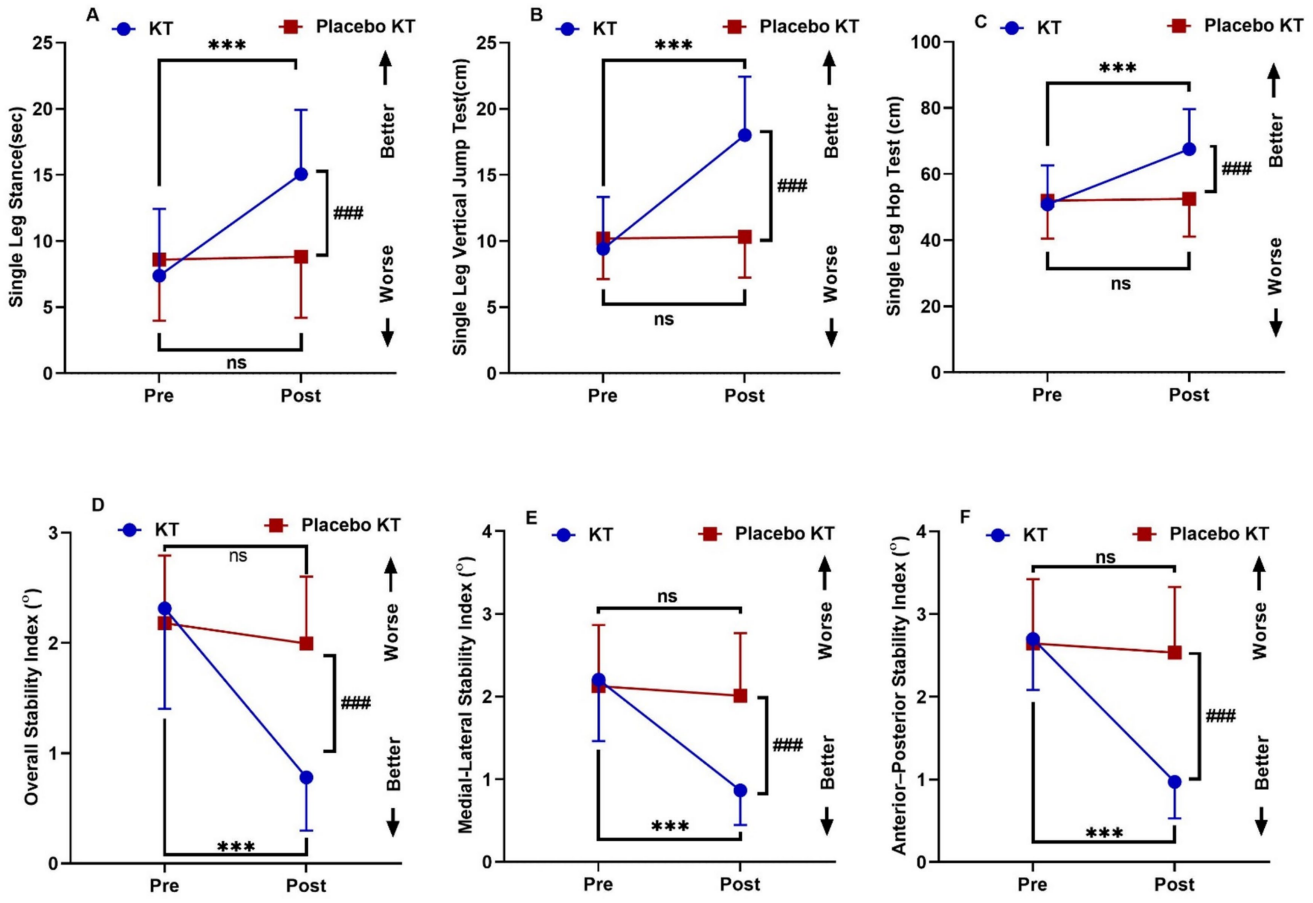
Interactions between group and time for **(A)** Single Leg Stance (sec) **(B)** Single Leg Vertical Jump Test (cm) **(C)** Single Leg Hop Test (cm) **(D)** Overall Stability Index **(E)** Medio-Lateral Stability Index **(F)** Antero-Posterior Stability Index. Data are expressed as mean ± SD. ^***^p < 0.001 within KT group pre *vs.* post-intervention, ^###^*p* < 0.001 of KT *vs.* KT placebo group post-intervention, ns: non-significant.

Post-intervention, there was a significant increase in SLS, SLVJT, and SLHT, while OSI, MLSI, and APSI exhibited a significant reduction in the KT group compared to pre-treatment values (*p* < 0.001). The SLS, SLVJT, SLHT, OSI, MLSI, and APSI scores did not differ significantly in the Placebo KT group. No significant differences were observed between the pre- and post-intervention groups for any of the variables. After the intervention, the mean values of the OSI, MLSI, and APSI in the KT group significantly decreased (*p* < 0.001) compared with those in the two group, whereas the SLS, SLVJT, and SLHT groups showed a significant increase in the KT group compared with the Placebo KT group (*p* < 0.001) (Table 2; Figure 4). A two-way repeated-measures ANOVA showed significant changes in SLS, SLVJT, SLHT, OSI, MLSI, and APSI between the KT and Placebo KT post-intervention, as evidenced by an effect size (η^2^) > 0.14. Post-hoc power analysis comparing the KT and placebo KT groups revealed a power of > 98% for all clinical measures.

**Table 2:** Clinical characteristics of subjects in both groups after 6 weeks of intervention (*N* = 40).

Variable	KT group				Placebo KT group				Group × time interaction (F) *p* value	*P^b^* between groups	Effect size (η2)	*Post-hoc power analysis
	Pre	Post	MD (95%CI)	*P^a^* within group	Pre	Post	MD (95%CI)	*P^a^* within group				(1-β err prob) (%)
SLS (sec)	7.37 ± 5.07	15.07 ± 4.86	7.69 (4.51–10.87)	<0.001	8.60 ± 4.63	8.83 ± 4.62	0.23 (−2.73–3.18)	0.88	*F*_1, 38_ = 10.69	<0.001	0.54	0.982 (98.2%)
									<0.01			
SLVJT (cm)	9.41 ± 3.92	18.01 ± 4.42	8.60 (5.92–11.28)	<0.001	10.19 ± 3.06	10.32 ± 3.09	0.13 (−1.83–2.10)	0.89	*F*_1, 38_ = 22.61	<0.001	0.42	>0.999 (>99.9%)
									<0.001			
SLHT (cm)	50.80 ± 11.84	67.51 ± 12.12	16.71 (9.04–24.38)	<0.001	51.92 ± 11.46	52.54 ± 11.41	0.61 (−6.71–7.93)	0.87	*F*_1, 38_ = 8.67	<0.001	0.51	0.977 (97.7%)
									<0.01			
OSI (°)	2.32 ± 0.91	0.78 ± 0.48	−1.54 (−2.00 to-1.07)	<0.001	2.18 ± 0.61	2.00 ± 0.61	−0.19 (−0.58–0.21)	0.34	*F*_1, 38_ = 30.74	<0.001	0.75	>0.999 (>99.9%)
									<0.001			
MLSI (°)	2.21 ± 0.74	0.87 ± 0.42	−1.34 (−1.73 to −0.95)	<0.001	2.13 ± 0.74	2.01 ± 0.76	−0.12 (−0.59–0.36)	0.63	*F*_1, 38_ = 33.35	<0.001	0.58	>0.999 (>99.9%)
									<0.001			
APSI (°)	2.70 ± 0.62	0.97 ± 0.44	−1.73 (−2.07 to −1.39)	<0.001	2.65 ± 0.78	2.54 ± 0.79	−0.11 (−0.61–0.39)	0.66	*F*_1, 38_ = 32.39	<0.001	0.79	>0.999 (>99.9%)
									<0.001			

SLS, Single Leg Stance; SLVT, Single Leg Vertical Jump Test; SLHT, Single Leg Hop Test; OSI, Overall Stability Index; MLSI, Medial-Lateral Stability Index; APSI, Anterior–Posterior Stability Index; Values are shown as mean ± standard deviation (SD). CI, Confidence Interval; MD, Mean Difference; *P^a^*, value within group from paired-samples *t*-test; *P^b^*, value post-intervention from independent-samples *t*-test; η^2^, partial eta square; group × time interaction *p* value from two-way repeated measures ANOVA.

*Post-hoc power analysis (1-β error probability) (%) between the KT and placebo KT groups post-intervention.

### Correlation between SLS, SLVJT, and SLHT and OSI

**Figure 5:**
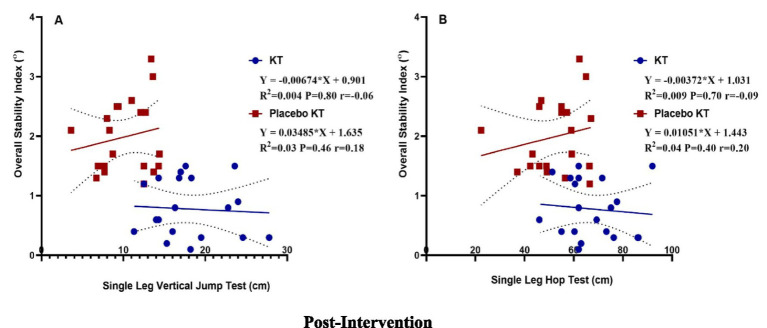
Post-intervention linear regression: **(A)** single-leg vertical jump test and overall stability index and **(B)** single-leg hop test and overall stability index.

Table 3 shows a weak positive correlation between the SLVJT and OSI tests in the Placebo KT group (*r* = 0.18), but a negative correlation in the KT group (*r* = −0.06). Linear regression analysis showed no statistically significant differences. The increased SLVJT explained a 0.04% decrease in OSI in the KT group and a 3% increase in the KT placebo group post-intervention (Figure 5A). There was a weak positive correlation between SLHT and OSI in the KT placebo group (*r* = 0.20) but a negative correlation in the KT group (*r* = −0.09). Linear regression analysis showed non-significant results. In the KT placebo group, an increase in SLHT was associated with a 4% increase in the OSI. In contrast, an increase in SLHT in the KT group was related to a 0.09% decrease in OSI (Figure 5B) post-intervention.

**Table 3:** Factors associated with the overall stability index in the KT group *vs.* the placebo KT group post-intervention.

Regression statistics	Single leg vertical jump test		Single leg hop test	
	KT group	Placebo KT group	KT group	Placebo KT group
Correlation coefficient (*r*)	−0.06	0.18	−0.09	0.20
*R* ^2^	0.004	0.03	0.009	0.04
Adjusted *R*^2^	−0.05	−0.02	−0.05	−0.01
Regression parameters				
B (95% CI)	−0.01 (−0.06, 0.05)	0.03 (−0.06 to 0.13)	–0.004 (−0.02, 0.02)	0.01 (−0.02 to 0.04)
Beta	−0.06	0.18	−0.09	0.20
SE	0.03	0.05	0.009	0.01
*t*-value	−0.26	0.76	−0.40	0.86
*P*-value	0.80 (ns)	0.46 (ns)	0.70 (ns)	0.40 (ns)

*r*, Pearson coefficient; *R*^2^, R square coefficient of determination; B, unstandardized coefficient estimate; Beta, standardized coefficient estimate; SE, standard error of the coefficient estimate; CI, confidence interval; *p* ≤ 0.05 is statistically significant; ns, non-significant.

## Discussion

The main results of the current study revealed that ankle KT significantly improved static and dynamic balance and functional performance when compared to placebo KT in obese female university participants (*p* < 0.001). The present study was conducted on female individuals only, as it is suggested that women have a 25% greater chance of sustaining ankle sprains (Grade I), which is the most common injury, than their male counterparts (25). Additionally, ankle instability may not be linked to the injury because the ligaments that connect the ankle bones are only microscopically stretched rather than ruptured (26). Given that women often have higher laxity in their ankle joints, it is plausible that KT improves stability in women. Males also have more active muscle stiffness than females, which increases their resistance to changes in muscle length, suggesting that males have more stable joints. Thus, it is possible that females with poorer joint stability benefit more from tactile input (27).

The main results of the current study showed that ankle KT resulted in a statistically significant increase in SLS and a significant decrease in OSI, MLSI, and APSI compared with the placebo KT group (*p* < 0.001). Because static and dynamic balance are modulated by various mechanisms, they cannot be coupled; hence, they were assessed separately (28).

Kinesio taping (KT) is believed to improve balance through several physiological mechanisms. KT primarily enhances proprioceptive feedback by stimulating cutaneous mechanoreceptors in the skin, thereby increasing sensory input to the central nervous system and improving joint position sense, a crucial component of balance control (29). Additionally, tape can facilitate or inhibit muscle activity depending on its direction and tension, promoting optimal muscle coordination and stability (16). KT also serves as a tactile cue, helping individuals maintain or correct postural alignment, which reduces compensatory movements that can impair balance (30). Furthermore, by enhancing joint stability and reducing pain through mechanisms such as gate control theory, KT indirectly supports improved balance by enabling more natural and confident movement patterns (31). These combined effects make KT a potentially useful adjunct in balance rehabilitation, particularly in populations with proprioceptive or neuromuscular deficits (32).

The findings of the current study are consistent with those of previous studies (11, 33–38). KT improves acute postural control in healthy athletes and those with chronic ankle instability (CAI) (11). Moreover, KT applied to the foot improves dynamic postural stability in healthy athletes (34). Static and dynamic balance improved after the application of KT combined with dynamic taping for ankle sprains in patients with CAI (35). Moreover, OSI, MLSI, and APSI improved following the combination of KT and exercises for ankle instability in recreational runners (36). Additionally, in patients with first-degree ankle sprains, KT improved dynamic postural control, as evaluated using the Star Excursion Balance Test (SEBT), more than no tape and athletic tape (33). Furthermore, ankle stability, as well as static and dynamic balance, improved in participants with ankle instability as a result of the mechanical enhancement of the muscles and compression of the joint by flexible taping (37). Six weeks of KT combined with balance exercises enhanced the balance and stability scores of female athletic participants (38).

KT has an immediate effect on dynamic balance and postural stability, attributed to KT’s elastic nature, which may regulate balance by promoting proprioception around the ankle joint (39). The potential benefits of KT on dynamic balance were only obvious with prolonged tape application (after 24 h) in women (40).

In contrast to the present study results, some studies have revealed no beneficial efficacy of KT mechanical correction techniques on dynamic postural control using different taping materials (41, 42). This contradiction may be attributed to the fact that the injuries of individuals with CAI result from a lack of proprioception brought on by central mechanisms or by using different balancing techniques, compensating for employing more proximal (hip) than ankle strategies during static balance (41). Additionally, using computerized dynamic posturography, Mulligan ankle taping was found to have no discernible effect on postural and motor control in healthy participants (42). Other studies on the immediate effect of KT on balance (43–45) contradicted the results of the present study. KT has a limited ability to improve postural stability in patients with persistent ankle sprains (43). This may be because the KT was not sufficiently taut to support the deep sensory receptors found in the tendon organs and muscle spindles. Similarly, there was no discernible variation in balance or functional performance after applying KT to the calf muscle (44). These results may be attributed to the different populations, including healthy active individuals, and various KT application techniques and tensions (KT applied with 50% tension on the gastrocnemius muscle starting from the origin and ending at the insertion). Additionally, KT did not improve static or dynamic balance compared to no tape or placebo (45). This may be due to the short duration of the study and the different application techniques used in the study. Moreover, taping did not improve postural control during sagittal and frontal plane landing tasks in subjects with CAI (46). This contradiction might be due to differences in sample size and measurement tools.

In addition, KT did not improve the sense of stability in athletes with ankle instability (47). This difference from the current finding may be related to the different population, sample size, and technique of application, as it was applied as a single strip from the origin to the insertion of the fibularis longus muscle.

For participants with CAI, no significant change in dynamic postural stability was observed after the application of either taping mechanism (lateral subtalar sling or fibular repositioning) (41). This contradiction may be due to the small sample size (only 16 participants) and the different application techniques used.

In terms of functional performance, the present study revealed a significant increase in SLVJT and SLHT in the KT group compared with the Placebo KT group (*p* < 0.001). This notable effect might result from the stimulatory activity of KT on different sensory receptors on the skin, which enhances the capacity of the muscles to contract, thereby increasing the function of the muscles and joints and enhancing muscle power and gait (48). KT is clinically helpful in the management of lateral ankle injuries as it improves proprioception, muscular endurance, and activity performance (49). It has been proposed that improving the mobility and stability of the ankle and foot enhances the ability to jump and land (50).

The conclusions of the current study are in line with prior studies (39, 51–54). KT is efficient in improving ankle functional performance in healthy individuals (51, 53, 54). Furthermore, it may improve the strength and performance of male semi-professional soccer players with and without FAI, as measured using the hop test immediately after its application (39, 52).

In contrast to the current results, previous studies (55, 56) found a non-significant effect of KT on functional performance. In young, healthy individuals, ankle KT does not affect vertical jump height (55). This discrepancy may be attributed to variations in application techniques, KT tension, or patient populations. Moreover, KT applied to a healthy population found no improvement or worsening of the hop test (56). A possible explanation for this contradiction might be the differing characteristics of the populations, as well as the different treatment durations.

Additionally, in healthy athletes, KT application did not immediately promote functional performance, as evaluated by the highest vertical jump performance and peak jump power in the vertical jump test (57). This contradiction might be due to differences in the population, application techniques, and treatment duration.

The effect of taping versus preventive bracing techniques on functional balance and jumping performance of physically active male university students was evaluated, and there were no significant effects between the modalities (58), possibly due to differences in measurement tools.

The findings of the current study showed a weak positive correlation between the SLVJT and OSI tests in the Placebo KT group (*r* = 0.18), whereas a negative correlation was observed in the KT group (*r* = −0.06). In addition, there was a weak positive correlation between SLHT and OSI in the KT placebo group (*r* = 0.20), but a negative correlation in the KT group (*r* = −0.09). Given that dynamic balance describes the capacity to sustain a steady center of gravity throughout periods of movement, such as hop distance, it seems to be one of the most important physical-functional aspects affecting SLHT values (59).

In line with the present study results, KT showed a statistically significant negative correlation with the single-leg Balance Error Scoring System (BESS) score and vertical jumping performance (*r* = −0.596) in football players (60). Moreover, it has been proven that balance training boosts jump height and that jump training enhances balance performance (61). Similarly, balance training has been shown to improve agility and vertical jump scores in physical education students and those who participate in hands-on recreational activities (62). Furthermore, it contributes to improved performance in single-leg hop tests in young elite female basketball players (59). Additionally, increased dynamic stability enhances one-legged hop-landing biomechanics (63).

## Implications for rehabilitation

- Rehabilitation professionals should implement mechanical correction techniques for ankle KT as an effective strategy to improve balance and functional performance in obese female university students in the future.
- The mechanical correction technique of ankle KT should be tailored to address the specific needs of young obese females to enhance musculoskeletal health, reduce the risk of falls, and limit ankle injury risk.
- The mechanical correction technique of ankle KT provides a safe and non-pharmacological adjuvant therapy for enhancing balance and musculoskeletal performance in obese females.

## Limitations and recommendations

This study had several limitations that warrant consideration. This study included only healthy obese females. Future research should ideally include studies involving both sexes. Furthermore, this study lacked long-term follow-up evaluations, which makes it desirable for future research to conduct long-term follow-up studies to address this limitation.

## Conclusion

Ankle Kinesio taping, using the mechanical correction technique for six weeks, can improve static and dynamic balance, as well as functional performance in obese female university students, making it a beneficial addition to rehabilitation programs targeting balance impairments in this population.

## Data Availability

The raw data supporting the conclusions of this article will be made available by the authors, without undue reservation.

## References

[1] Zamora-Kapoor A Sinclair K Nelson L Lee H Buchwald D. Obesity risk factors in American Indians and Alaska natives: a systematic review. Public Health. (2019) 174:85–96. doi: 10.1016/j.puhe.2019.05.021, PMID: 31326761

[2] Chooi YC Ding C Magkos F. The epidemiology of obesity. Metabolism. (2019) 92:6–10. doi: 10.1016/j.metabol.2018.09.005, PMID: 30253139

[3] Capodaglio P Gobbi M Donno L Fumagalli A Buratto C Galli M. Effect of obesity on knee and ankle biomechanics during walking. Sensors (Basel). (2021) 21:7114. doi: 10.3390/s21217114, PMID: 34770421 PMC8588043

[4] Brockett CL Chapman GJ. Biomechanics of the ankle. Orthop Trauma. (2016) 30:232–8. doi: 10.1016/j.mporth.2016.04.015, PMID: 27594929 PMC4994968

[5] Hou Q. Biomechanics of the ankle: exploring structure, function, and injury mechanisms. Stud Sports Sci Phys Educ. (2023) 1:1–16. doi: 10.56397/ssspe.2023.09.01

[6] Yamazaki T Maruyama S Sato Y Suzuki Y Shimizu S Kaneko F. A preliminary study exploring the change in ankle joint laxity and general joint laxity during the menstrual cycle in cis women. J Foot Ankle Res. (2021) 14:21–1. doi: 10.1186/s13047-021-00459-7, PMID: 33761990 PMC7988940

[7] Lee H Yim J. Increased postural sway and changes in the neuromuscular activities of the ankle stabilizing muscles at ovulation in healthy young women. Tohoku J Exp Med. (2016) 240:287–94. doi: 10.1620/tjem.240.287, PMID: 27941275

[8] Bravi R Cohen E Quarta E Martinelli A Minciacchi D. Effect of direction and tension of Kinesio taping application on sensorimotor coordination. Int J Sports Med. (2016) 37:909–14. doi: 10.1055/s-0042-109777, PMID: 27454132

[9] Magalhães I Bottaro M Freitas JR Carmo J Matheus JPC Carregaro RL. Prolonged use of Kinesiotaping does not enhance functional performance and joint proprioception in healthy young males: randomized controlled trial. Braz J Phys Ther. (2016) 20:213–22. doi: 10.1590/bjpt-rbf.2014.0151, PMID: 27437712 PMC4946837

[10] Li R Qin R Tan Y Liu H Wang K Cheng L. Effect of kinesio taping intervention on the muscle strength and balance of college basketball players with functional ankle instability. Front Physiol. (2023) 14:1064625–5. doi: 10.3389/fphys.2023.1064625, PMID: 37064886 PMC10090455

[11] Someeh M Norasteh AA Daneshmandi H Asadi A. Immediate effects of mulligan's fibular repositioning taping on postural control in athletes with and without chronic ankle instability. Phys Ther Sport. (2015) 16:135–9. doi: 10.1016/j.ptsp.2014.08.003, PMID: 25165014

[12] Tomruk MS Tomruk M Alkan E Gelecek N. Is ankle Kinesio taping effective to immediately change balance, range of motion, and muscle strength in healthy individuals? A randomized, sham-controlled trial. Korean J Fam Med. (2022) 43:109–16. doi: 10.4082/kjfm.21.0015, PMID: 35320896 PMC8943236

[13] Erdeo F Uca AU Çankaya M Yılmaz NA. Does kinesio taping affect balance in individuals with multiple sclerosis? Neurol Sci. (2025) 46:3183–90. doi: 10.1007/s10072-025-08115-9, PMID: 40131653 PMC12152069

[14] Nunes GS Feldkircher JM Tessarin BM Bender PU da Luz CM de Noronha M. Kinesio taping does not improve ankle functional or performance in people with or without ankle injuries: systematic review and meta-analysis. Clin Rehabil. (2020) 35:182–99. doi: 10.1177/0269215520963846, PMID: 33081510

[15] Chaabene H Negra Y Capranica L Bouguezzi R Hachana Y Rouahi MA. Validity and reliability of a new test of planned agility in elite taekwondo athletes. J Strength Cond Res. (2018) 32:2542–7. doi: 10.1519/jsc.0000000000002325, PMID: 29120989

[16] Kase K Wallis J Kase T. Clinical therapeutic applications of the Kinesio taping® method. Dallas: Kinesio Taping Association International (2013).

[17] Inglés M Serra-Añó P Méndez ÀG Zarzoso M Aguilar-Rodríguez M Suso-Martí L. Effect of Kinesio taping and balance exercises on postural control in amateur soccer players: a randomised control trial. J Sports Sci. (2019) 37:2853–62. doi: 10.1080/02640414.2019.1677016, PMID: 31613172

[18] Springer BA Marin R Cyhan T Roberts H Gill NW. Normative values for the Unipedal stance test with eyes open and closed. J Geriatr Phys Ther. (2007) 30:8–15. doi: 10.1519/00139143-200704000-00003, PMID: 19839175

[19] Dawson N Dzurino D Karleskint M Tucker J. Examining the reliability, correlation, and validity of commonly used assessment tools to measure balance. Health Sci Rep. (2018) 1:e98–8. doi: 10.1002/hsr2.98, PMID: 30623052 PMC6295615

[20] Hussein HM. Postural indices and limits of stability in subjects having chronic low back pain versus healthy control: a cross-sectional comparative study. Rehabil Med. (2021) 25:25. doi: 10.5604/01.3001.0015.2424, PMID: 40693429

[21] Lee DW Yang SJ Cho SI Lee JH Kim JG. Single-leg vertical jump test as a functional test after anterior cruciate ligament reconstruction. Knee. (2018) 25:1016–26. doi: 10.1016/j.knee.2018.07.014 30115591

[22] Arjang N MohsenİFar H AmİRİ A Dadgoo M RasaeİFar G. The acute effect of static versus proprioceptive neuromuscular facilitation stretching combined with kinesiology taping® of hamstring muscles on functional tests in adolescent taekwondo athletes. Türk Fizyoterapi ve Rehabilitasyon Dergisi. (2023) 34:21–8. doi: 10.21653/tjpr.974941

[23] Sullivan SW Fleet NA Brooks VA Bido J Nwachukwu BU Brubaker PH. Comparison of different functional tests for leg power and normative bilateral asymmetry index in healthy collegiate athletes. Open Access J Sports Med. (2021) 12:119–28. doi: 10.2147/OAJSM.S315162, PMID: 34393525 PMC8354771

[24] MedCalc Software Ltd. Power estimator for comparison of means. Version 23.2.6. Available online at: https://www.medcalc.org/calc/power-comparison-of-means.php (Accessed June 15, 2025).

[25] Talia AJ Busuttil NA Kendal AR Brown R. Gender differences in foot and ankle sporting injuries: a systematic literature review. Foot. (2024) 60:102122. doi: 10.1016/j.foot.2024.102122, PMID: 39121692

[26] Hutson M Ward A. Oxford textbook of musculoskeletal medicine. London, UK: Oxford University Press (2015).

[27] Trevino J Lee H. Sex differences in 2-DOF human ankle stiffness in relaxed and contracted muscles. Ann Biomed Eng. (2018) 46:2048–56. doi: 10.1007/s10439-018-2092-9, PMID: 30003504

[28] Pau M Arippa F Leban B Corona F Ibba G Todde F. Relationship between static and dynamic balance abilities in Italian professional and youth league soccer players. Phys Ther Sport. (2015) 16:236–41. doi: 10.1016/j.ptsp.2014.12.003, PMID: 25869425

[29] Halseth T McChesney JW DeBeliso M Vaughn R Lien J. The effects of kinesio™ taping on proprioception at the ankle. J Sports Sci Med. (2004) 3:1–7. PMID: 24497814 PMC3896108

[30] Jaraczewska E Long C. Kinesio® taping in stroke: improving functional use of the upper extremity in hemiplegia. Top Stroke Rehabil. (2006) 13:31–42. doi: 10.1310/33KA-XYE3-QWJB-WGT6 16987790

[31] Lee J-H Yoo W-G Lee K-S. Effects of head-neck rotation and kinesio taping of the flexor muscles on dominant-hand grip strength. J Phys Ther Sci. (2010) 22:285–9. doi: 10.1589/jpts.22.285

[32] Aytar A Ozunlu N Surenkok O Baltacı G Oztop P Karatas M. Initial effects of kinesio® taping in patients with patellofemoral pain syndrome: a randomized, double-blind study. Isokinet Exerc Sci. (2011) 19:135–42. doi: 10.3233/IES-2011-0413

[33] Mohamed MA Radwan NL Azab ASR. Effect of kinesio-taping on ankle joint stability. Int J Med Res Health Sci. (2016) 5:51–8. Available at: https://www.ijmrhs.com/medical-research/effect-of-kinesiotaping-on-ankle-joint-stability.pdf

[34] Fouladi R Veleshkolai SKH Abaee M Bahnamiri FJ. Kinesio taping and dynamic postural stability in different foot posture. Physiotherapy. (2015) 101:e405. doi: 10.1016/j.physio.2015.03.634

[35] Lim J-s Kim S-h Moon I-y Yi C-h. The effects of elastic ankle taping on static and dynamic postural control in individuals with chronic ankle instability. Phys Ther Korea. (2021) 28:200–7. doi: 10.12674/ptk.2021.28.3.200

[36] Hussein HM Kamel WM Kamel EM Attyia MR Acar T Kanwal R. The effect of kinesio taping on balance and dynamic stability in college-age recreational runners with ankle instability. Healthcare (Basel). (2023) 11:1749. doi: 10.3390/healthcare11121749, PMID: 37372867 PMC10297994

[37] Kim H-S Park J-Y. Effect of muscle taping and joint taping on static and dynamic balance in normal adults with chronic ankle instability. J Korean Soc Integr Med. (2022) 10:101–8. doi: 10.15268/ksim.2022.10.1.101

[38] Khalili SM Barati AH Oliveira R Nobari H. Effect of combined balance exercises and kinesio taping on balance, postural stability, and severity of ankle instability in female athletes with functional ankle instability. Life (Basel). (2022) 12:178. doi: 10.3390/life12020178, PMID: 35207466 PMC8879431

[39] Lee B-G Lee J-H. Immediate effects of ankle balance taping with kinesiology tape on the dynamic balance of young players with functional ankle instability. Technol Health Care. (2015) 23:333–41. doi: 10.3233/thc-150902, PMID: 25735310

[40] Akbari A Sarmadi A Zafardanesh P. The effect of ankle taping and balance exercises on postural stability indices in healthy women. J Phys Ther Sci. (2014) 26:763–9. doi: 10.1589/jpts.26.763, PMID: 24926148 PMC4047248

[41] Delahunt E McGrath A Doran N Coughlan GF. Effect of taping on actual and perceived dynamic postural stability in persons with chronic ankle instability. Arch Phys Med Rehabil. (2010) 91:1383–9. doi: 10.1016/j.apmr.2010.06.023, PMID: 20801256

[42] de-la- Morena JMD Alguacil- Diego IM Molina-Rueda F Ramiro-González M Villafañe JH Fernández-Carnero J. The mulligan ankle taping does not affect balance performance in healthy subjects: a prospective, randomized blinded trial. J Phys Ther Sci. (2015) 27:1597–602. doi: 10.1589/jpts.27.1597 26157271 PMC4483449

[43] Yin L Wang L. Acute effect of kinesiology taping on postural stability in individuals with unilateral chronic ankle instability. Front Physiol. (2020) 11:192–2. doi: 10.3389/fphys.2020.00192, PMID: 32265726 PMC7105687

[44] Wilson V Douris P Fukuroku T Kuzniewski M Dias J Figueiredo P. The immediate and long-term effects of kinesiotape® on balance and functional performance. Int J Sports Phys Ther. (2016) 11:247–53. PMID: 27104058 PMC4827367

[45] Esposito F Barni L Manzi F Braccio P Latella L Corvi A. Does ankle Kinesio taping® application improve static and dynamic balance in healthy trained semi-professional soccer male players? A single blinded randomized placebo controlled crossover study. Sci Sports. (2021) 36:e167–74. doi: 10.1016/j.scispo.2021.02.002

[46] De Ridder R Willems T Vanrenterghem J Roosen P. Effect of tape on dynamic postural stability in subjects with chronic ankle instability. Int J Sports Med. (2015) 36:321–6. doi: 10.1055/s-0034-1385884, PMID: 25665000

[47] Briem K Eythörsdöttir H Magnúsdóttir RG Pálmarsson R Rúnarsdöttir T Sveinsson T. Effects of kinesio tape compared with nonelastic sports tape and the untaped ankle during a sudden inversion perturbation in male athletes. J Orthop Sports Phys Ther. (2011) 41:328–35. doi: 10.2519/jospt.2011.3501, PMID: 21212501

[48] Kim M-K Cha H-G. The effects of ankle joint taping on gait and balance ability of healthy adults. J Phys Ther Sci. (2015) 27:2913–4. doi: 10.1589/jpts.27.2913, PMID: 26504323 PMC4616124

[49] Wilson B Bialocerkowski A. The effects of kinesiotape applied to the lateral aspect of the ankle: relevance to ankle sprains--a systematic review. PLoS One. (2015) 10:e0124214-e0124214. doi: 10.1371/journal.pone.0124214, PMID: 26103637 PMC4477981

[50] Patti A Gervasi M Giustino V Figlioli F Canzone A Drid P. The influence of ankle mobility and foot stability on jumping ability and landing mechanics: a cross-sectional study. J Funct Morphol Kinesiol. (2024) 9:160. doi: 10.3390/jfmk9030160, PMID: 39311268 PMC11417945

[51] Lee S-M Lee J-H. The immediate effects of ankle balance taping with kinesiology tape on ankle active range of motion and performance in the balance error scoring system. Phys Ther Sport. (2017) 25:99–105. doi: 10.1016/j.ptsp.2016.08.013, PMID: 28117264

[52] Fereydounnia S Shadmehr A Attarbashi Moghadam B Talebian Moghadam S Mir SM Salemi S. Improvements in strength and functional performance after Kinesio taping in semi-professional male soccer players with and without functional ankle instability. Foot. (2019) 41:12–8. doi: 10.1016/j.foot.2019.06.006, PMID: 31675595

[53] Wang Y Gu Y Chen J Luo W He W Han Z. Kinesio taping is superior to other taping methods in ankle functional performance improvement: a systematic review and meta-analysis. Clin Rehabil. (2018) 32:026921551878044–1481. doi: 10.1177/0269215518780443, PMID: 30020820

[54] Hong S Lee E-H Eom H-R Yeom C-K Park S-Y Jeong Y-B. Effect of ankle taping on the ankle muscle strength in young healthy women. J Hum Sport Exerc. (2020) 15:15. doi: 10.14198/jhse.2020.152.20

[55] Nakajima MA Baldridge C. The effect of kinesio® tape on vertical jump and dynamic postural control. Int J Sports Phys Ther. (2013) 8:393 24175126 PMC3812836

[56] Fayson SD Needle AR Kaminski TW. The effects of ankle Kinesio® taping on ankle stiffness and dynamic balance. Res Sports Med. (2013) 21:204–16. doi: 10.1080/15438627.2013.792083, PMID: 23777376

[57] Cheung R Yau Q Wong K Lau P So A Chan N. Kinesiology tape does not promote vertical jumping performance: a deceptive crossover trial. Man Ther. (2016) 21:89–93. doi: 10.1016/j.math.2015.06.001, PMID: 26139360

[58] Ozer D Senbursa G Baltaci G Hayran M. The effect on neuromuscular stability, performance, multi-joint coordination and proprioception of barefoot, taping or preventative bracing. Foot. (2009) 19:205–10. doi: 10.1016/j.foot.2009.08.002 20307478

[59] Dominguez-Navarro F Casaña J Perez-Dominguez B Ricart-Luna B Cotolí-Suárez P Calatayud J. Dynamic balance and explosive strength appears to better explain single leg hop test results among young elite female basketball athletes. Sci Rep. (2023) 13:5476–6. doi: 10.1038/s41598-023-31178-7, PMID: 37016001 PMC10073233

[60] Erkmen N Taşkin H Sanioğlu A Kaplan T Baştürk D. Relationships between balance and functional performance in football players. J Hum Kinet. (2010) 26:21–9. doi: 10.2478/v10078-010-0044-z

[61] Goktepe M Gunay M Bezci S Bayram M Ozkan A. Correlations between different methods of vertical jump and static balance parameters in athletes. Turk J Sport Exerc. (2016) 18:147. doi: 10.15314/tjse.42907

[62] Hrysomallis C. Balance ability and athletic performance. Sports Med. (2011) 41:221–32. doi: 10.2165/11538560-000000000-00000 21395364

[63] Boey D Jc LM. The relationship between y-balance test scores and knee moments during single-leg jump-landing in netball. Int J Sports Phys Ther. (2020) 15:722–31. doi: 10.26603/ijspt20200722, PMID: 33110691 PMC7575156

